# Supplementary material for: TPGS-based and S-thanatin functionalized nanorods for overcoming drug resistance in Klebsiella pneumonia
Source: Nat Commun. 2022 Jun 29;13:3731. doi: 10.1038/s41467-022-31500-3 (PMC9243133; doi:10.1038/s41467-022-31500-3)
Supplement: Supplementary file 1 — Supplementary Information [file 41467_2022_31500_MOESM1_ESM.pdf]

Supplementary Information

TPGS-based and S-thanatin functionalized nanorods for overcoming drug resistance in *Klebsiella pneumonia*

Xiaojuan Wang[1], Xiaoling Xu[2], Shaojun Zhang[1], Na Chen[1], Yunfeng Sun[2], Kuifen Ma[1], Dongsheng Hong[1], Lu Li[1], Yongzhong Du[2*], Xiaoyang Lu[1*], Saiping Jiang[1*]

[1] Department of Clinical Pharmacy, The First Affiliated Hospital, School of Medicine, Zhejiang University, 79 Qingchun Road, Hangzhou 310003, China

[2] Institute of Pharmaceutics, College of Pharmaceutical Sciences, Zhejiang University, 866 Yu-Hang-Tang Road, Hangzhou, 310058, China

Correspondence:

Saiping Jiang, E-mail: j5145@zju.edu.cn

Xiaoyang Lu, E-mail: luxiaoyang@zju.edu.cn

Yongzhong Du, E-mail: duyongzhong@zju.edu.cn

Content **Page**

Supplementary Fig. 1 The inhibitory effect of TPGS on efflux pumps. **S3**

Supplementary Fig. 2 Fluorescence images of EA.hy926 cells after incubation with NT-TPGS/Cap and Ts-TPGS/Cap nanorods. **S4**

Supplementary Fig. 3 The quantitative analysis of bacterial counts in the BALF of KPN-infected and TRKP-infected pneumonia mice **S5**

Supplementary Table 1. FIC indices against KPN and TRKP. **S6**

Supplementary Table 2. Primer sequences for efflux pump genes **S6**

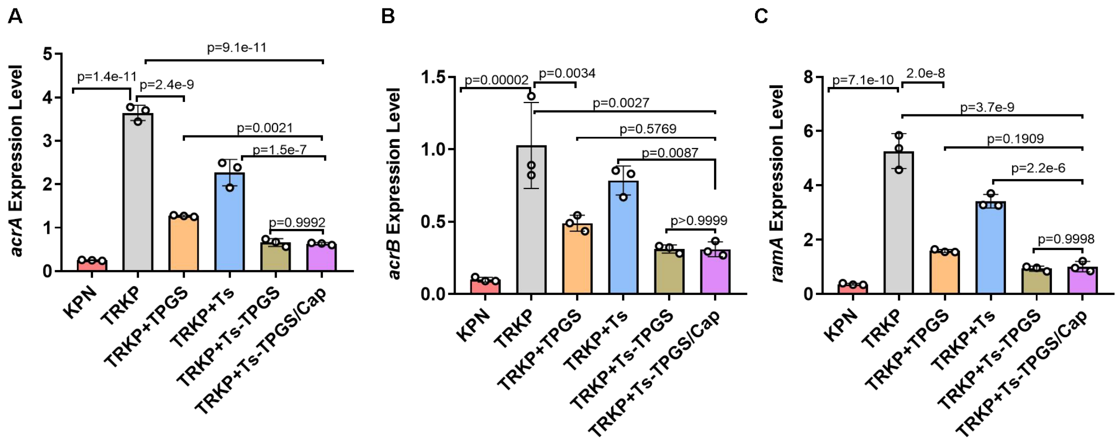

Supplementary Fig.1 The inhibitory effect of TPGS on efflux pumps. The expression levels of (A) *acrA*, (B) *acrB* and (C) *ramA* of TPGS, Ts, Ts-TPGS and Ts-TPGS/Cap nanorods treated TRKP by RT-PCR method. KPN and TRKP without any treatment were used as control. Ts: S-thanatin peptide; TPGS: tocopheryl polyethylene glycol succinate; Ts-TPGS/Cap: Ts-TPGS functionalized calcium phosphate nanorods; KPN: *Klebsiella pneumonia*; TRKP: tigecycline-resistant *Klebsiella pneumonia.* Data are presented as the mean ± SD (n=3 independent experiments). One-way analysis of variance (ANOVA) with post hoc Tukey tests were performed in A, B and C.

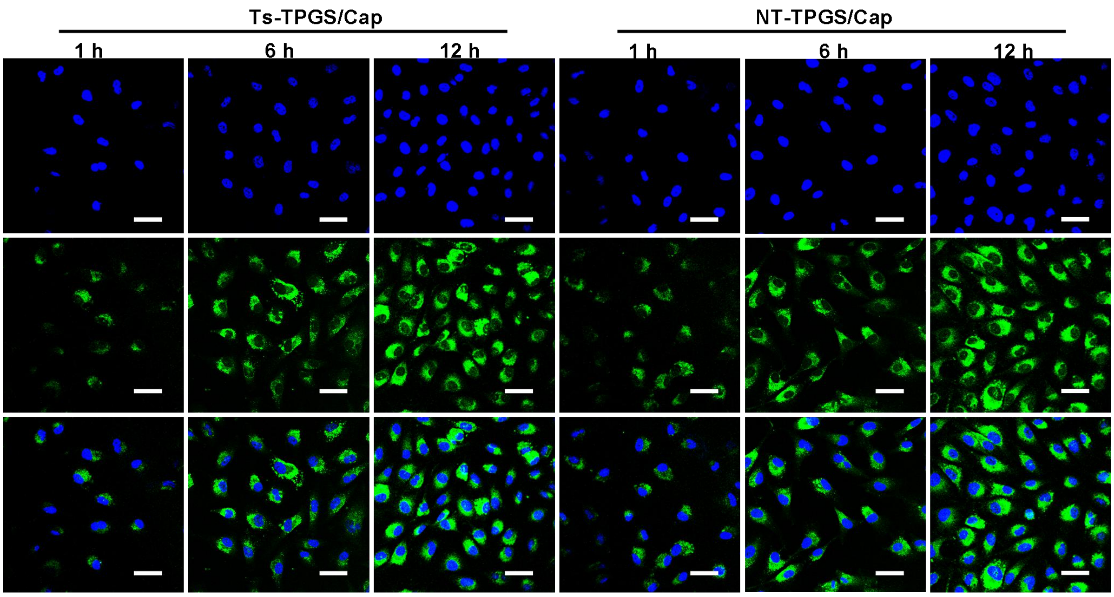

Supplementary Fig.2 Fluorescence images of EA.hy926 cells after incubation with NT-TPGS/Cap and Ts-TPGS/Cap nanorods. The cells were labeled with DAPI (blue), and the green fluorescence signal indicated the nanorods. Scale bar = 50 μm. Ts-TPGS/Cap: Ts-TPGS functionalized calcium phosphate nanorods; NT-TPGS/Cap: non-targeting peptide-TPGS functionalized calcium phosphate nanorods. The experiments were repeated independently for three times with similar results.

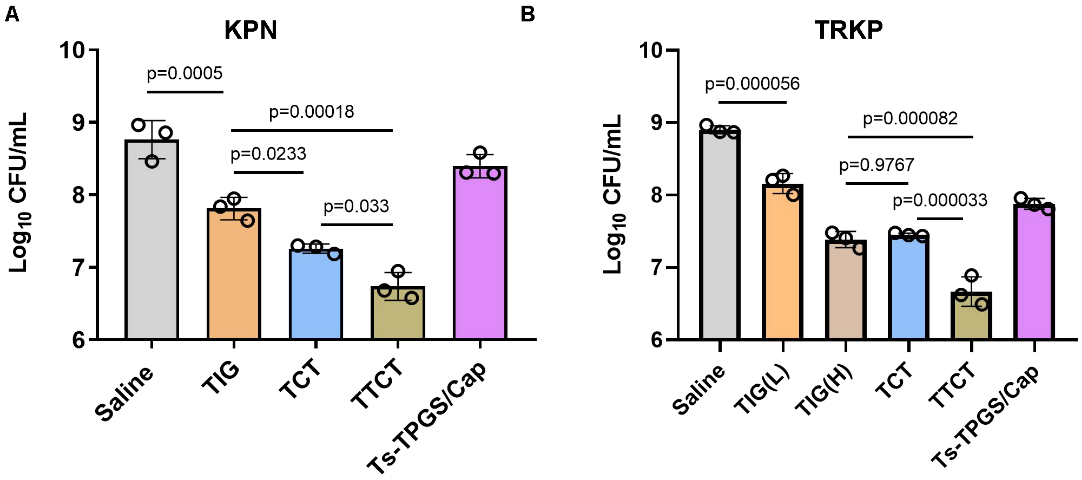

Supplementary Fig.3 The quantitative analysis of bacterial counts in the BALF of KPN-infected (A) and TRKP-infected pneumonia mice after 48 h receiving various treatments. TIG: free tigecycline; TIG(L): low dose of tigecycline (15 mg/kg); TIG (H): high dose of tigecycline (45 mg/kg); TCT: tigecycline loaded TPGS/Cap nanorods; TTCT: tigecycline-loaded Ts-TPGS/Cap nanorods; KPN: *Klebsiella pneumonia*; TRKP: tigecycline-resistant *Klebsiella pneumonia;* BALF: bronchoalveolar lavage fluid. Data are presented as the mean ± SD (n = 3 mice). One-way analysis of variance (ANOVA) with post hoc Tukey tests were performed in A and B.

Supplementary Table 1. FIC indices against KPN and TRKP

Microorganisms	Combination	MIC (ug/mL)		FICI	Interpretation
		Individual	Combined		
TRKP	TIG	4	1	0.375	synergistic
	Ts	16	2		
KPN	TIG	1	0.5	0.75	additive
	Ts	16	4		

Supplementary Table 2. Primer sequences for efflux pump genes

Primer name	Primer Sequence
acrA-F	ATGTGACGATAAACCGGCTC
acrA-R	CTGGCAGTTCGGTGGTTATT
acrB-F	CGATAACCTGATGTACATGTCC
acrB-R	CCGACAACCATCAGGAAGCT
ramA-F	GCATCAACCGCTGCGTATT
ramA-R	GGGTAAAGGTCTGTTGCGAAT
16s-rRNA-F	CAGCTCGTGTCGTGAGATGT
16s-rRNA-R	CGTAAGGGCCATGATGACTT
