# Supplementary material for: TPGS-based and S-thanatin functionalized nanorods for overcoming drug resistance in Klebsiella pneumonia
Source: Nat Commun. 2022 Jun 29;13:3731. doi: 10.1038/s41467-022-31500-3 (PMC9243133; doi:10.1038/s41467-022-31500-3)
Supplement: Supplementary file 3 — Reporting Summary [file 41467_2022_31500_MOESM3_ESM.pdf]

nature portfolio

Corresponding author(s): Yongzhong Du, Xiaoyang Lu, Saiping Jiang

Last updated by author(s): June 12, 2022

Reporting Summary

Nature Portfolio wishes to improve the reproducibility of the work that we publish. This form provides structure for consistency and transparency in reporting. For further information on Nature Portfolio policies, see our Editorial Policies and the Editorial Policy Checklist.

Statistics

For all statistical analyses, confirm that the following items are present in the figure legend, table legend, main text, or Methods section.

n/a	Confirmed	
☐	☒	The exact sample size (*n*) for each experimental group/condition, given as a discrete number and unit of measurement
☐	☒	A statement on whether measurements were taken from distinct samples or whether the same sample was measured repeatedly
☐	☒	The statistical test(s) used AND whether they are one- or two-sided<br>*Only common tests should be described solely by name; describe more complex techniques in the Methods section.*
☒	☐	A description of all covariates tested
☒	☐	A description of any assumptions or corrections, such as tests of normality and adjustment for multiple comparisons
☐	☒	A full description of the statistical parameters including central tendency (e.g. means) or other basic estimates (e.g. regression coefficient) AND variation (e.g. standard deviation) or associated estimates of uncertainty (e.g. confidence intervals)
☒	☐	For null hypothesis testing, the test statistic (e.g. *F*, *t*, *r*) with confidence intervals, effect sizes, degrees of freedom and *P* value noted<br>*Give P values as exact values whenever suitable.*
☒	☐	For Bayesian analysis, information on the choice of priors and Markov chain Monte Carlo settings
☒	☐	For hierarchical and complex designs, identification of the appropriate level for tests and full reporting of outcomes
☒	☐	Estimates of effect sizes (e.g. Cohen's *d*, Pearson's *r*), indicating how they were calculated

Our web collection on statistics for biologists contains articles on many of the points above.

Software and code

Policy information about availability of computer code

Data collection	The NMR spectra were obtained using an NMR spectrometer (AC-80, BrukerBioSpin, Germany). Fluorescence intensity was measured on a fluorescence spectrophotometer (F-2500, Hitachi High-Technologies Co., Japan). Transmission electron microscopy (TEM) images were acquired on an electron microscopy (JEOL JEM-1230, Japan). Dynamic light scattering (DLS) data were determined by Malvern ZetaSizer Nano instrument. Flow cytometry data were acquired from ACEA NovoCyte. Confocal laser scanning microscopy (CLSM) images were obtained on Flowview FV3000 and Flowview FV3000 microscopy. Biodistribution images of mice were acquired on IVIS Spectrum imaging system (PerkinElmer, USA). PET-CT images were obtained by a Siemens Inveon combined micro PET-CT scanner (Siemens Preclinical Solution USA, Inc., Knoxville, TN, USA)
Data analysis	MestReNova 6.1.1/Graphpad Prism 7.0/Microsoft Excel/FlowJo_V10/NovoExpress

For manuscripts utilizing custom algorithms or software that are central to the research but not yet described in published literature, software must be made available to editors and reviewers. We strongly encourage code deposition in a community repository (e.g. GitHub). See the Nature Portfolio guidelines for submitting code & software for further information.

Data

Policy information about availability of data

All manuscripts must include a data availability statement. This statement should provide the following information, where applicable:

- Accession codes, unique identifiers, or web links for publicly available datasets
- A description of any restrictions on data availability
- For clinical datasets or third party data, please ensure that the statement adheres to our policy

The authors declared that the main data supporting the findings are available within the article and its Supplementary Information. Extra data are available from the corresponding authors upon reasonable request. Source data are provided with this paper.

Field-specific reporting

Please select the one below that is the best fit for your research. If you are not sure, read the appropriate sections before making your selection.

☒ Life sciences ☐ Behavioural & social sciences ☐ Ecological, evolutionary & environmental sciences

For a reference copy of the document with all sections, see nature.com/documents/nr-reporting-summary-flat.pdf

Life sciences study design

All studies must disclose on these points even when the disclosure is negative.

Sample size	Sample sizes used in this study were informed by previous studies performed in the investigators' laboratories or by other previously published studies that have used similar methods. Sample citations include:<br>(1) Wang, Xiao-Juan, et al. "Polysialic-acid-based micelles promote neural regeneration in spinal cord injury therapy." Nano letters 19.2 (2019): 829-838.<br>(2) Wang, Xiao-Juan, et al. "Combinational protective therapy for spinal cord injury medicated by sialic acid-driven and polyethylene glycol based micelles." Biomaterials 217 (2019): 119326.<br>(3) Kang, Xu-Qi, et al. "Effective targeted therapy for drug-resistant infection by ICAM-1 antibody-conjugated TPGS modified β-Ga2O3: Cr3+ nanoparticles." Theranostics 9.10 (2019): 2739.<br>(4) Kang, Xu-Qi, et al. "Tocopherol polyethylene glycol succinate-modified hollow silver nanoparticles for combating bacteria-resistance." Biomaterials science 7.6 (2019): 2520-2532.
Data exclusions	No data were excluded from the analysis.
Replication	All experiments were performed a minimum of three replicates in independent experiments with similar results. All attempts at replication were successful.
Randomization	Cells in each experiment were from the same pool of parental cells. KPN and TRKP in each experiment were from the same pool of parental bacteria. Mice were randomly allocated into cages before treated with corresponding test agents. KPN and TRKP-infected mice were randomly sorted into groups.
Blinding	For microscopy software, flow cytometry, animal IVIS systems, and other data collected by objective instruments, the investigators were not blinded to group allocation during data collection. But the investigator was blinded at the time of data analysis. The laboratory personnel who performed mice experiments was not blinded because they needed to know how to treat mice with different strategies. But the laboratory personnel was blinded during the data analysis from each individual mice.

Reporting for specific materials, systems and methods

We require information from authors about some types of materials, experimental systems and methods used in many studies. Here, indicate whether each material, system or method listed is relevant to your study. If you are not sure if a list item applies to your research, read the appropriate section before selecting a response.

Materials & experimental systems

n/a	Involved in the study	
☐	☒	Antibodies
☐	☒	Eukaryotic cell lines
☒	☐	Palaeontology and archaeology
☐	☒	Animals and other organisms
☒	☐	Human research participants
☒	☐	Clinical data
☒	☐	Dual use research of concern

Methods

n/a	Involved in the study	
☒	☐	ChIP-seq
☐	☒	Flow cytometry
☒	☐	MRI-based neuroimaging

Antibodies

Antibodies used	anti-myeloperoxidase (MPO) antibody (Abcam, ab208670)<br>PE-labeled anti-Ly-6G/Ly-6C antibody (eBioscience™ :12-5931-82)<br>FITC-labeled anti-CD11b antibody (eBioscience™: 11-0112-41)
Validation	Validation data of the antibodies purchased from commercial vendors are available on the manufactures' website and data sheets.<br>anti-myeloperoxidase (MPO) antibody: https://www.abcam.com/myeloperoxidase-antibody-epr20257-ab208670.html<br>PE-labeled anti-Ly-6G/Ly-6C antibody: https://www.thermofisher.cn/antibody/product/12-5931-82.html?CID=AFLLS-12-5931-82<br>FITC-labeled anti-CD11b antibody: https://www.thermofisher.cn/cn/zh/antibody/product/CD11b-Antibody-clone-M1-70-Monoclonal/11-0112-41

Eukaryotic cell lines

Policy information about cell lines

Cell line source(s)	EA.hy926 (catalog number: GNHu39) was obtained from Chinese Academy of Sciences Cell Bank (Shanghai, China). Human umbilical vein endothelial cells (HUVECs) was provided by Cell Resource Center,IBMS, CAMS/PUMC (catalog number: 1101HUM-PUMC000437)
Authentication	All cell lines were authenticated using STR profiling and species identifications.
Mycoplasma contamination	All cell lines were negative for mycoplasma contamination.
Commonly misidentified lines<br>(See ICLAC register)	None

Animals and other organisms

Policy information about studies involving animals; ARRIVE guidelines recommended for reporting animal research

Laboratory animals	Male ICR mice (6 to 8 weeks old, 22 - 25 g) were provided by Zhejiang Medical Animal Center and had free access to food and water.
Wild animals	None
Field-collected samples	None
Ethics oversight	The surgical procedures and in vivo experiments were conducted in compliance with the National Institutes of Health Guide for the Care and Use of Laboratory Animals and were approved by the Animal Care and Use Committee of the First Affiliated Hospital, College of Medicine, Zhejiang University.

Note that full information on the approval of the study protocol must also be provided in the manuscript.

Flow Cytometry

Plots

Confirm that:

- [x] The axis labels state the marker and fluorochrome used (e.g. CD4-FITC).
- [x] The axis scales are clearly visible. Include numbers along axes only for bottom left plot of group (a 'group' is an analysis of identical markers).
- [x] All plots are contour plots with outliers or pseudocolor plots.
- [x] A numerical value for number of cells or percentage (with statistics) is provided.

Methodology

Sample preparation	KPN and TRKP after different treatments were washed with PBS, fixed with 4% paraformaldehyde and analyzed by flow cytometry. KPN-infected and TRKP-infected mice were treated by various preparations. Then, bronchoalveolar lavage fluid (BALF) was collected at 24 h, and the cell pellet were harvested by centrifuging the BALF (1000 rpm, 10 min). After staining with PE-labeled anti-Ly-6G/Ly-6C and FITC-labeled anti-CD11b antibodies, the ratios of neutrophils in total cells were detected by flow cytometry.
Instrument	ACEA NovoCyte
Software	NovoExpress/FlowJo_V10
Cell population abundance	10000 cells in gate

Gating strategy

Cells were first gated by FSC-H and SSC-H, followed by FSC-H and FSC-A to obtain the single cell populations. Then the positive and negative populations with respective fluorochrome were gated.

☐ Tick this box to confirm that a figure exemplifying the gating strategy is provided in the Supplementary Information.
